# Thoughts of death or suicidal ideation are common in young people aged 12 to 30 years presenting for mental health care

**DOI:** 10.1186/1471-244X-12-234

**Published:** 2012-12-26

**Authors:** Elizabeth M Scott, Daniel F Hermens, Sharon L Naismith, Django White, Bradley Whitwell, Adam J Guastella, Nick Glozier, Ian B Hickie

**Affiliations:** 1 Clinical Research Unit, Brain & Mind Research Institute, University of Sydney, 100 Mallet Street, Camperdown, NSW 2050, Australia; 2 School of Medicine, Sydney, The University of Notre Dame, 160 Oxford St, Darlinghurst, 2010, Australia

**Keywords:** Suicide, Clinical staging, Psychiatric, Youth

## Abstract

**Background:**

Reducing suicidal behaviour is a major public health goal. Expanding access to care has been identified as a key strategy. In Australia, a national network of primary-care based services (*headspace*) has been established for young people with mental ill-health. This study determines the socio-demographic, psychopathological and illness-stage correlates of suicidal ideation in young persons attending *headspace* services.

**Methods:**

Suicidal ideation was recorded using the specific suicide item of the Hamilton Depression Rating Scale (HDRS) in a cohort of subjects aged 12-30 years (N = 494) attending *headspace* services.

**Results:**

Of the 494 young persons assessed, 32% (158/494) had a positive response to any level of the HDRS suicide item, consisting of 16% (77/494) reporting that life was not worth living and a further 16% (81/494) reported thoughts of death or suicidal ideation. Young women (19%; 94/494) were more likely to report any positive response as compared with young men (13%; 64/494) [χ^2^(2,494) = 13.6, p < .01]. Those with ‘attenuated syndromes’ reported positive responses at rates comparable to those with more established disorders (35% vs. 34%; χ^2^(1,347) = 0.0, p = 0.87). However, more serious levels of suicidal ideation were more common in those with depressive disorders or later stages of illness. In multivariate analyses, the major predictors of the degree of suicidal ideation were increasing levels of clinician-rated depressive symptoms (beta = 0.595, p < .001), general psychopathology (beta = 0.198, p < .01), and self-reported distress (beta = 0.172, p < .05).

**Conclusions:**

Feelings that life is not worth living, thoughts of death or suicidal ideation are common in young people seeking mental health care. These at-risk cognitions are evident before many of these individuals develop severe or persistent mental disorders. Thoughts of death or suicidal ideation may well need to be a primary intervention target in these young people.

## Background

While approximately six per cent of deaths globally in those aged 10-24 years are due to suicide [1], attempted suicide and related injuries also contribute significantly to the overall burden of illness in young people [2-4]. Suicide increases significantly after the age of 15 years and continues to rise in early adulthood [3]. Previous Australian data suggested that increasing access to treatments might have reduced completed suicides in the late 1990s [5]. While a range of population-based strategies are relevant for suicide prevention, enhanced access to quality services for young people with mental disorders has been prioritised [3].

In Australia, however, access to appropriate mental health care is less likely to occur in males and young people, and is also adversely affected by lower socio-economic status and living outside of the major metropolitan centres [6-8]. Previous studies have indicated the extent to which a variety of factors including how young people perceive common disorders like depression and suitability of available services or treatments [9], or how young people interact with primary care services [7], affect the probability that young people will use services or receive appropriate mental health care.

The development of the new national youth mental health services framework, *headspace*[10,11], has as one of its goals easier and earlier access to care for young people who are experiencing mental ill-health. Specifically, it is designed to attract those who may be at high risk - such as young men with concurrent alcohol or other substance misuse or those who might be experiences thoughts of self-harm or suicide. The aims of this study are to assess data collected from a large, representative *headspace* population and to evaluate the relationships between self-reported suicidal ideation and key risk factors, such as gender and substance use. Additionally, the potential relationships between suicidal risk factors and other demographic, diagnostic or illness-stage variables have not been explored in this unique youth cohort who are presenting for care early in the course of mental ill-health.

## Methods

The study and consent procedure was approved by the University of Sydney Human Research Ethics Committee (USYD HREC). All participants were determined by their referring clinician or mental health professional to have the mental and intellectual capacity to give written informed consent prior to participation in the study. Participants aged 16 years or older were able to give their own written informed consent (i.e. parental/guardian consent is not required for those aged 16 and above according to the USYD HREC guidelines and Australian law) prior to participation in the study. Parental (or guardian) written consent was obtained for subjects under 16 years of age.

### Participants

Four-hundred and ninety-four outpatients were recruited from two *headspace* services that specialise in the assessment and early intervention of mental health problems in young people [11,12]. Importantly, the key inclusion criteria for this study were not focused on issues related to self-harm or suicidal behaviour. The two key inclusion criteria were: (i) persons aged 12 to 30 years seeking professional help primarily for anxiety, depressive and/or psychotic symptoms that were associated with self-reported impairments in role function; and, (ii) willingness to participate in more detailed clinical, neurobiological and longitudinal research related to illness outcomes in young people [13,14]. Participants were consecutively recruited between October 2007 and December 2011; and were excluded if they had insufficient fluency in the English language to participate in the neuropsychological assessment (not reported here), were intellectually impaired (e.g. IQ < 70) or had current substance dependence. Participants were asked to abstain from drug and alcohol use for 48 hours prior to testing. Referring clinicians were asked to determine primary and secondary diagnoses based on DSM-IV criteria. For the purposes of this study, patients were then grouped into the relevant broad categories (primary diagnoses) of ‘depression’, ‘bipolar’, ‘psychosis’, and ‘other’ (which included anxiety-, behavioural-, autistic spectrum-, learning-, substance use-, and personality- disorders).

### Clinical assessment

Following earlier clinical assessment and active illness management by the referring clinician (i.e. a psychiatrist, clinical psychologist or general practitioner with training in mental health), a trained research psychologist conducted a structured clinical interview in this sub-group of subjects to record details of the nature and history of any mental health problems. As a proxy measure for duration of illness, the age that each patient first engaged a mental health service was recorded. The interviewer completed the Brief Psychiatric Rating Scale (BPRS; [15]) to quantify general psychopathology and the Hamilton Depression Rating Scale (HDRS) [16] to rate severity of depressive symptoms. The third item of the HDRS was utilised to determine the level of a patient’s current suicidal thoughts or ideation. Specifically the item was asked as follows: “This past week, have you had any thoughts that life is not worth living? What about thinking you’d be better off dead? Have you had thoughts of hurting or killing yourself?” (If answered “yes”, a further prompt was: “What have you thought about? Have you actually done anything to hurt yourself?”). Ratings were then based on the following scores: 0 = ‘absent’; 1 = ‘feels life is not worth living’; 2 = ‘wishes s/he were dead or any thoughts of possible death to self’; 3 = ‘suicidal ideas or gestures’ and 4 = ‘attempts at suicide (any serious attempt)’. The social and occupational functioning assessment scale (SOFAS; [17]) was also used as a rating of the patient’s functioning from 0 to 100, with lower scores indicating more severe impairment.

### Clinical staging

Our clinical staging model [18] builds on routine clinical assessment (though it may be assisted by ancillary investigations). A stage of illness is formally assigned at the end of the clinical assessment phase. Such clinical assessment captures: (i) current major symptoms (severity, frequency, type); (ii) characteristic mental features; (iii) age of onset and clinical course of illness prior to presentation; (iv) previous “worst ever” symptoms and treatments including hospital admissions; (v) current level of risks of harm due to illness; (vi) previous suicide attempts or other at-risk behaviours; and, (vii) current (as compared with premorbid) levels of social, educational or employment functioning. Once this information is obtained and integrated, a clinical stage is assigned according to sets of established criteria (see [18]). As described in detail elsewhere [18], our staging model includes five discrete categories: stage 1a = ‘help-seeking’; stage 1b = ‘attenuated syndrome’; stage 2 = ‘discrete disorder’; stage 3 = ‘recurrent or persistent disorder’; and stage 4 = ‘severe, persistent and unremitting illness’.

Importantly, these stages do not use the same thresholds for disorder as DSM or ICD-defined anxiety, mood or psychotic disorders. In previous work [11], we have demonstrated that many single episode or uncomplicated DSM-defined anxiety or depressive disorders are staged as 1a or 1b respectively, while established psychotic, bipolar or severe depressive disorders are likely to be rated as stage 2 or above (depending on other illness course variables).

### Self report

Participants completed self-report measures that included the Kessler-10 (K-10) [19] which is a brief instrument designed to detect severity of general psychological distress and the Alcohol Use Disorders Identification Test (AUDIT) to assess each participant’s level of risky drinking in the past year, as well their lifetime familiarity [20,21].

### Statistical analyses

Performed using SPSS for Windows 20.0; group differences in demographic, clinical and self report variables were assessed via ANOVA or chi-square tests where relevant. If equality of variance was compromised (according to Levene’s test) the corrected degrees of freedom and p-values were reported. Scheffé’s tests were used to determine post-hoc pair-wise comparisons. To identify the relative significance of any distinguishing variables (or their pattern of co-association) of illness factors that were significant in the univariate analyses were entered into a final multiple stepwise regression model.

## Results

Among the 494 young persons assessed, 15.6% (77/494) reported that life was not worth living, while 16.4% (81/494) reported thoughts of death or suicidal ideation (scored as 2 or more on the relevant HDRS item); resulting in a total of 31.9% (158/494) reporting a positive response at any level of the HDRS suicide item. Notably, the proportion of positive responders was comparable in both adolescents (12 to 17 years) and young adults (18 to 30 years) at 30.3% (51/168) and 32.8% (107/326), respectively. Across the entire sample (i.e. 12 to 30 years), females (19.0%; 94/494) were more likely to report any positive response as compared with males (12.9%; 64/494) [χ^2^(2,494) = 13.6, p < .01].

As outlined in Table 1, those reporting at-risk phenomena were more likely to be female, assessed by clinicians to be functioning more poorly and as being more severely depressed or having more behavioural disturbance, and to be self-reporting more general distress. Higher levels of alcohol use were not a strong predictor of thoughts of death or suicidal ideation in this cohort. A stepwise multiple regression using the five significant variables (i.e. gender, SOFAS, HDRS total, BPRS total, K10 total; see Table 1) as predictors of thoughts of death or suicidal ideation revealed a three-step model: [R^2^ = 0.351 for step 1, change in R^2^ = 0.019 for step 2 (p < .01), change in R^2^ = 0.015 for step 3 (p < .05)]. In step one, only depressive symptoms (total HDRS; beta = 0.595, p < .001) were included in the model; in step two, behavioural disturbance (total BPRS; beta = 0.198, p < .01) was added; and in step 3, self-reported distress (total K10; beta = 0.172, p < .05) contributed to the overall model (R^2^ = 0.388). That is, both clinician-rated and self-reported symptom severities, rather than other demographic or comorbid features, emerged as strong predictors of thoughts of death or suicidal ideation in this cohort.

**Table 1:** Demographic and clinical correlates of suicidal ideation in young people presenting for mental health care

	***No suicidal ideation (n = 336)***^***a***^	***“Life not worth living” (n = 77)***^***b***^	***Thoughts of death or Suicidal ideation (n = 81)***^***c***^	***Significance Test [p]***	***Post hoc***		
					**a vs. b**	**a vs. c**	**b vs. c**
Females, n (%)	145 (43%)	41 (53%)	53 (65%)	χ^2^ (2, 493) = 13.6 [.001]	na	na	na
Age, years	19.9 ± 4.6	19.4 ± 4.1	20.0 ± 4.1	F (2, 493) = 0.4 [.706]			
Age of onset, years	15.4 ± 4.7	15.4 ± 4.8	14.1 ± 3.7	F (2, 397) = 2.5 [.086]			
Predicted IQ	100.6 ± 11.7	101.4 ± 9.2	102.9 ± 9.5	F (2, 147.6) = 1.6 [.212]			
Education, years	11.5 ± 2.8	11.5 ± 2.5	11.5 ± 2.5	F (2, 489) = 0.1 [.942]			
SOFAS	62.4 ± 12.0	58.0 ± 11.2	57.0 ± 11.7	F (2, 387) = 7.2 [.001]	*	**	
HDRS total	8.7 ± 5.7	14.8 ± 5.6	19.0 ± 5.6	F (2, 476) = 120.7 [.000]	***	***	***
BPRS total	36.7 ± 8.6	43.7 ± 9.8	49.2 ± 10.5	F (2, 475) = 67.8 [.000]	***	***	***
K10 total	23.0 ± 7.4	30.4 ± 7.8	33.5 ± 6.1	F (2, 424) = 73.8 [.000]	***	***	*
AUDIT total	6.9 ± 7.7	7.5 ± 8.6	8.2 ± 8.5	F (2, 491) = 0.9 [.399]			

**Note**: Final columns provide the results of the post-hoc Scheffe’s pairwise comparisons (with corresponding significance levels: * < .05; ** p < .01; *** p < .001) where ‘a’ denotes the “No suicidal ideation” group (i.e. HDRS item-3 score = 0); ‘b’ denotes the “Life not worth living” group (i.e. HDRS item-3 score = 1) and ‘c’ denotes the “Thoughts of death or Suicidal ideation” group (i.e. HDRS item-3 score >1). SOFAS = Social and Occupational Functioning Assessment Scale; HDRS = Hamilton Depression rating Scale; BPRS = Brief Psychiatric Rating Scale; K-10 = Kessler-10; AUDIT = Alcohol Use Disorders Identification Test.

The prevalence of individuals reporting any symptom on the suicide item (i.e. ‘life not worth living’ or ‘thoughts of death’ or ‘suicidal ideation’) versus those who didn’t (i.e. ‘absent’) was significantly different across the four diagnostic groups [χ^2^(3, 494) = 14.7, p < .01] (see Table 2). Notably, those with depressive disorders reported the highest rates of any positive response to the suicide item (41.9%; 78/186). Similarly, the rates of those reporting thoughts of death or suicidal ideation (i.e. a score of 2 or more versus 1 or below) was also significantly different across the diagnostic groups [χ^2^(3, 494) = 13.4, p < .01], with the depression group having approximately twice the rate of reporting the more serious symptoms compared to the remaining three diagnostic groups.

**Table 2:** Diagnostic and illness-stage correlates of suicidal ideation in young people presenting for mental health care

	**% [n]**			
	**No suicidal ideation**	**Any suicidal ideation**	***“Life not worth living”***	***Thoughts of death or Suicidal ideation***
*Diagnostic category*				
Depression	58.1 [108]	41.9 [78]	17.7 [33]	24.2 [45]
Bipolar	69.6 [55]	30.4 [24]	20.3 [16]	10.1 [8]
Psychosis	74.4 [90]	25.6 [31]	13.2 [16]	12.4 [15]
Other	76.9 [83]	23.1 [25]	11.1 [12]	12.0 [13]
Clinical Stage*				
Stage 1a: help-seeking	83.3 [60]	16.7 [12]	9.7 [7]	6.9 [5]
Stage 1b: attenuated syndromes	64.9 [155]	35.1 [84]	19.7 [47]	15.5 [37]
Stage 2: discrete disorder	65.7 [71]	34.3 [37]	12.0 [13]	22.2 [24]
Stage 3+: recurrent or persistent major illness	66.1 [41]	33.9 [21]	12.9 [8]	21.0 [13]

**Note**: “No suicidal ideation” denotes HDRS item-3 score = 0; “Any suicidal ideation” denotes HDRS item-3 > 0; “Life not worth living” denotes HDRS item-3 score = 1; “Thoughts of death or Suicidal ideation” denotes HDRS item-3 score >1. HDRS = Hamilton Depression rating Scale. *Clinical stage data was missing for N = 13 cases. Chi-square testing for differences in rates of ‘no’ versus ‘any’ suicidal ideation was significantly different across diagnostic groups [χ^2^(3, 494) = 14.7, p < .01] as well as illness stage [χ^2^(3, 481) = 9.2, p < .05].

With regards to illness stage, there were significant differences in the rates of individuals reporting any symptom on the suicide item [χ^2^(3, 481) = 9.2, p < .05] and in the rates of those who reported the more severe thoughts of death or suicidal ideation [χ^2^(3, 481) = 8.4, p < .05] (see Table 2). Approximately 35% of those at stage 1b or greater reported any positive response to the HDRS suicide item. The lack of major difference (i.e. 35.1% vs. 34.3%; [χ^2^(1,347) = 0.0, p = 0.87]) between those classed as being at stage 1b (i.e. attenuated syndromes) versus stage 2/3 (discrete or established disorders) is notable. Within the 1b category (i.e. below our threshold for established or persistent disorders), 15.5% of subjects reported overt suicidal ideation (37/239). Even among those rated as being at the earliest stage of illness (1A) the prevalence of any positive response to the HDRS suicide item was almost 17%.

## Discussion

Almost a third of young people attending youth-orientated mental health services and participating in longitudinal research report some degree of suicidal ideation (32%%). Such thoughts are well-accepted risk factors for later acts of self-harm or overt suicidal behaviour [3]. Although many of these individuals do not yet have established mental disorders, the rates of these ‘at-risk’ phenomena are much higher than in the general population – where cumulative rates of up to 10% over the entire adolescent or early adult period may be expected [3].

Even among those classed as being at the earliest or least severe phase (stage 1a) of mental ill-health, 17% report any positive symptom on the suicide-related item. These prevalence rates rise sharply in those with ‘attenuated syndromes’ (stage 1b, approximately 35%). It is possible that rates are even higher in those with more established disorders who present to other more specialised mental health or emergency forms of care. Consistent with that view, there was a strong relationship in our cohort between severity of depressive and other behavioural symptoms and degree of suicidal ideation. Indeed, more serious levels of suicidal ideation were more common in those with depressive disorders or later stages of illness.

In this group of young people, however, it is clear that thoughts of death or suicidal ideation do occur early in the course of illness and are not simply restricted to those with more persistent or severe mental disorders. Importantly, these data are consistent with the view that thoughts of death or overt suicidal ideation may be strong drivers to seeking health-care, independent of other illness-related factors. This would be consistent with the literature that is available about attempted suicide [22]. Help-care seeking may be occurring either directly or through the intervention of other third parties. Certainly, the role of parents, friends and other key persons in assisting young people to come to care is evident in other data we have reported from these service sites [11].

While conventional diagnostic systems draw strong links between depressive disorders and thoughts of death or overt suicidal ideation, it is clear in this cohort that such phenomena do occur frequently across the diagnostic groupings examined. This may reflect the fact that such diagnostic groupings are not yet well differentiated in young persons [18] or the reality that thoughts of death or overt suicidal ideation are common across most mental disorders. Importantly, however, formal depressive disorders and more severe disorders of all types were associated with more overt suicidal ideation or behaviour (see Table 2).

The data set reported here has important limitations. It relies on identification of thoughts of death or suicidal ideation at systematic evaluation on entry to more detailed neurobiological studies rather than at the initial clinical assessment. While this may have biased the study towards inclusion of more severe or persistent cases (with consequent higher rates of thoughts of death or suicidal ideation) it also would have favoured the inclusion of those young people with more persistent thoughts of death or overt suicidal ideation. Importantly, the demographic characteristics of this subset of subjects, and the distribution of illness-stage and diagnoses, closely match the wider cohort [10,11]. Although we did not record respondent rates (i.e. the proportions of subjects who entered our services but refused to participate in the neurobiological studies) the current sample is representative of a larger cohort of N = 1260 subjects we have reported on previously [11], who had similar levels of functioning and psychological distress. Additionally, the recording of suicide-related phenomena on one occasion (via a Hamilton depression item) may differ from recordings that are done on more than one occasion.

## Conclusions

The relatively high rate of thoughts of death or overt suicidal ideation indicates that this health-care pathway attracts a population that is at much higher risk of suicide or self-harm than young people in the general population [4,23], who tend not have adequate contact with specialised mental health services [23]. While the general goal of *headspace* services is to attract young people at risk of poor long-term outcomes, these data indicate that another specific need is readily apparent – namely, strategies to reduce thoughts of death or overt suicidal ideation. In conventional practice, it is often assumed that treatment of the primary mental disorder is the most efficient way to reduce risk of self-inflicted harm. This study suggests that direct management of thoughts of death, suicidal ideation or behaviour by appropriate psychological or behavioural strategies may well need to be the primary target – especially given the fact that many of these young people may not yet have reached conventional thresholds for initiating other illness-specific strategies.

## Abbreviations

ANOVA: Analysis of variance; AUDIT: Alcohol use disorders identification test; BPRS: Brief psychiatric rating scale; DSM: Diagnostic and statistical manual of mental disorders; HDRS: Hamilton depression rating scale; ICD: International classification of diseases; K10: Kessler-10; SOFAS: Social and occupational functioning assessment scale; SPSS:Statistical package for the social sciences.

## Competing interests

Elizabeth Scott is the Clinical Director of the *headspace* clinics at the Brain and Mind Research Institute. She has received honoraria for her contributions to professional educational seminars related to depression, youth mental health and circadian-rhythms research. She is a member of a national advisory board for desvenlafaxine, supported by Pfizer. She is a participant in studies evaluating the therapeutic actions of agomelatine, supported by Servier. Ian Hickie is supported by a National Health and Medical Research Council Australia Fellowship (No. 464914). He was a director of *headspace*: the national youth mental health foundation until January 2012. He is the executive director of the Brain and Mind Research Institute which operates two early-intervention youth services under contract to *headspace*. He is a member of the new Australian National Mental Health commission and was previously the CEO of beyondblue: the national depression initiative. He has led a range of community-based and pharmaceutical industry-supported depression awareness and education and training programs. He has led depression and other mental health research projects that have been supported by a variety of pharmaceutical partners. Current investigator-initiated studies are supported by Servier and Pfizer. He has received honoraria for his contributions to professional educational seminars related to depression, youth mental health and circadian-rhythms research.

## Authors’ contributions

EMS, IBH and DFH prepared the initial draft manuscript. EMS, BW and IBH supervised and verified all clinical assessments. DFH and DW conducted the statistical analyses. EMS, DFH, SN and IBH conceived the study design. SN, BW, AG and NG provided interpretation of the clinical data and participated in various aspects of the study design and data collection. All authors contributed significantly to the interpretation of the data as well as having read and approved the final manuscript.

## Pre-publication history

The pre-publication history for this paper can be accessed here:

http://www.biomedcentral.com/1471-244X/12/234/prepub
